# Proteome-Wide Reverse Vaccinology and Immunoinformatics-Guided Design of a Multi-Epitope Vaccine Against *Legionella pneumophila*

**DOI:** 10.3390/ijms27187977

**Published:** 2026-09-08

**Authors:** Lokesh Thangamani, Tong Lian

**Affiliations:** 1 Sanya Institute, Hainan Academy of Agricultural Sciences, Sanya 572000, China; 2 Institute of Crop Sciences, Chinese Academy of Agricultural Sciences, Beijing 100081, China; 3 National Nanfan Research Institute, Chinese Academy of Agricultural Sciences, Sanya 572000, China; 4 Institute of Food Crops Hainan Academy of Agricultural Sciences, Hainan Key Laboratory of Crop Genetics and Breeding, Haikou 571100, China

**Keywords:** *Legionella pneumophila*, multi-epitope vaccine, immunoinformatics, reverse vaccinology, molecular docking, molecular dynamics simulation

## Abstract

*Legionella pneumophila* is a Gram-negative opportunistic pathogen responsible for Legionnaires’ disease, a severe form of atypical pneumonia associated with high morbidity and mortality, particularly in immunocompromised individuals. Despite its growing global burden and the persistence of environmental reservoirs, no licensed vaccine is currently available. In this study, an integrative immunoinformatics and reverse vaccinology approach was employed to design a multi-epitope vaccine candidate targeting *L. pneumophila*. The complete proteome was systematically analyzed to identify essential, virulent, and surface-accessible proteins. Subsequent antigenicity assessment and non-homology screening against the human proteome led to the selection of key immunogenic targets, including KDO transferase and TolR. B-cell and T-cell epitopes were predicted and rigorously filtered based on antigenicity, non-allergenicity, and non-toxicity. Selected epitopes were assembled into a multi-epitope vaccine construct using appropriate linkers and adjuvants to enhance immunogenicity and structural stability. The designed construct exhibited favorable physicochemical properties, high antigenicity, and good solubility. Structural modeling and refinement confirmed the reliability of the predicted three-dimensional structure. Molecular docking analysis demonstrated strong binding interactions with immune receptors TLR-2 and TLR-9, which were further validated by molecular dynamics simulations indicating stable complex formation under physiological conditions. In addition, immune simulation predicted robust humoral and cellular immune responses, including memory cell generation. Overall, this study presents a promising computationally designed multi-epitope vaccine candidate against *L. pneumophila*. However, further in vitro and in vivo studies are required to validate its immunogenicity, safety, and protective efficacy.

## 1. Introduction

*Legionella pneumophila* is a ubiquitous environmental microorganism belonging to the family Legionellaceae, widely distributed in natural and artificial aquatic systems. It is a small, Gram-negative, aerobic, non-spore-forming bacillus that is typically catalase- and oxidase-positive, with a genomic guanine–cytosine (GC) content ranging between 38% and 52% [1]. Although generally described as motile, variations in flagellar expression among different serogroups result in heterogeneity in motility, precluding its strict classification as either motile or non-motile. The organism exhibits notable thermotolerance, proliferating optimally between 25 °C and 37 °C, while replication declines sharply at temperatures above 46 °C [2,3].

A defining ecological feature of *L. pneumophila* is its ability to persist in aquatic environments through surface attachment and biofilm formation. Within these structured microbial communities, the bacterium produces an extracellular polymeric matrix that enhances survival under nutrient-limited conditions and protects against environmental stressors. Biofilms also facilitate prolonged persistence, often spanning months to years, and promote horizontal gene transfer, which may contribute to the evolution of virulence traits. The regulation of biofilm formation has been linked to genetic determinants such as the bffA gene [4].

In humans, *L. pneumophila* is the primary etiological agent of legionellosis, a waterborne infectious disease that has emerged as a significant global public health concern. Despite being underdiagnosed and underreported, the incidence of legionellosis has shown a steady increase over recent decades. The disease manifests predominantly as Legionnaires’ disease (LD), a severe form of pneumonia, and accounts for substantial morbidity, mortality, and healthcare burdens [5,6]. Notably, *L. pneumophila* is responsible for over 90% of reported cases worldwide, with serogroup 1 alone contributing to approximately 80% of infections in Europe. Clinical severity often necessitates intensive care unit (ICU) admission in 20–40% of cases, particularly due to complications such as acute respiratory failure, septic shock, and renal dysfunction (Mondino et al., 2020) [7].

The economic burden of Legionnaires’ disease is considerable, ranking among the most costly waterborne illnesses, particularly in developed countries such as the United States. Hospitalization expenses per case can range widely, reflecting disease severity and associated complications [8]. Moreover, co-infections with viral pathogens (e.g., influenza viruses) or bacterial species such as *Mycoplasma pneumoniae*, *Chlamydia* spp., *Streptococcus pneumoniae*, *Klebsiella pneumoniae*, and *Pseudomonas aeruginosa* are frequently reported and are associated with worsened clinical outcomes, especially in immunocompromised individuals [9].

Clinically, the incubation period of Legionnaires’ disease typically ranges from 2 to 10 days following exposure to contaminated water or aerosols. Early manifestations include nonspecific symptoms such as fever, malaise, and fatigue, which may progress to a productive cough and signs of pulmonary involvement. Radiological findings are variable but commonly reveal patchy, unilateral infiltrates that can advance to consolidation. In addition to pulmonary symptoms, extrapulmonary manifestations, including gastrointestinal disturbances (nausea, vomiting, and diarrhea) and renal impairment, are frequently observed. Rare but severe complications such as myocarditis, neurological involvement, and multi-organ failure have also been documented [10].

Accurate diagnosis of legionellosis remains challenging due to its clinical similarity to other forms of community-acquired pneumonia. However, certain laboratory abnormalities, such as an elevated erythrocyte sedimentation rate (ESR), increased C-reactive protein (CRP), hyponatremia, and microscopic hematuria, may provide supportive evidence. Among diagnostic tools, urinary antigen testing using immunochromatographic methods is widely employed due to its rapid turnaround time and high specificity (>99%) and sensitivity (>85%) for detecting *L. pneumophila*, particularly serogroup 1 [11,12,13].

Therapeutic management of Legionnaires’ disease depends on disease severity. Current first-line treatments include macrolides, particularly azithromycin, and fluoroquinolones such as levofloxacin and moxifloxacin [14]. Mild to moderate cases are typically managed with short courses of oral azithromycin, whereas severe infections require intravenous administration of fluoroquinolones or macrolides. Combination therapy may be considered in critically ill patients. Notably, azithromycin demonstrates superior efficacy compared to other macrolides, which are generally bacteriostatic against *Legionella* species [15,16,17].

Recent evidence suggests that susceptibility to legionellosis may be exacerbated in patients with viral infections such as COVID-19, where factors including lymphopenia and immunosuppressive therapies (e.g., corticosteroids such as dexamethasone) may increase vulnerability. Established risk factors for severe disease include advanced age, smoking, chronic respiratory conditions, and immunosuppression due to underlying diseases or therapeutic interventions. Individuals with malignancies, diabetes, or renal disorders are particularly at risk, although the incidence does not appear to be disproportionately higher in patients with HIV/AIDS despite potentially increased disease severity [18,19,20].

Despite advances in antimicrobial therapy, no licensed vaccine is currently available for the prevention of legionellosis, and emerging antibiotic resistance poses an additional challenge. Consequently, there is an urgent need for novel preventive strategies. In this context, the present study employed an integrated proteome-wide reverse vaccinology and immunoinformatics workflow using the complete proteome of *Legionella pneumophila* subsp. *pneumophila* strain L01C.1 (Proteome ID: UP000277145). The proteome was systematically screened to identify essential, virulence-associated, surface-exposed, and antigenic proteins for the rational design of a multi-epitope vaccine candidate capable of eliciting both humoral and cellular immune responses.

## 2. Results

### 2.1. Protein Selection

The complete proteome of *Legionella pneumophila* subsp. *pneumophila* (L01C.1), consisting of 3218 proteins, was obtained from the UniProt database in FASTA format. Essential proteins were identified using the Geptop 2.0 server, resulting in a total of 403 essential proteins. Subcellular localization analysis with the PSORTb tool indicated that 87 essential proteins were distributed across key compartments, including the cytoplasmic membrane, periplasmic space, outer membrane, or extracellular region. The VIRULENTPRED server identified 88 virulent proteins, and the overlap between essential and virulent proteins yielded a final list of 11 promising candidates. These proteins are not only vital for bacterial survival but also contribute to immune evasion.

Transmembrane helices for these proteins were predicted using TMHMM v2.0, and molecular weights were determined using the ExPASy ProtParam tool. Immunogenic proteins were selected based on their ability to elicit effective immune responses. Antigenicity analysis, conducted with the Vaxijen 2.0 server at a threshold of 0.5, identified two proteins with high antigenicity scores. To prevent potential autoimmune responses, BLASTp analysis was performed against the human proteome, confirming no significant similarity for the selected proteins. These results support the identification of two promising vaccine candidates in Table 1. The detailed results of Geptop, PSORTb, and VIRULENTPRED, along with the analysis of the refined list, are in Supplementary File S1.

### 2.2. B-Cell and T-Cell Epitope Prediction

B-cell epitopes, both linear and conformational, were identified using the ABCpred server. Predicted epitopes with a fixed length of 14 amino acids were analyzed, and those with high scores were shortlisted based on their antigenicity, non-allergenicity, and non-toxicity. The top-ranked epitopes, listed in Table 2, were chosen for their immunogenic properties and suitability for further vaccine development. T-cell epitopes, including both MHC Class I and Class II, were identified using the IEDB tool. Epitopes with a percentile rank of ≤1.0 were selected, and peptides with lengths of nine amino acids were analyzed. The identified MHC Class I epitopes are expected to induce cytotoxic T lymphocyte (CTL) responses, which are crucial for recognizing and eliminating infected cells. Both MHC Class I and Class II epitopes exhibited low percentile ranks, indicating a strong binding affinity to their respective MHC molecules. The identified MHC Class II epitopes are detailed in Table 3 and Table 4.

### 2.3. Antigenicity, Allergenicity, and Toxicity Analysis

The selected B- and T-cell epitopes were further assessed for antigenicity using the Vaxijen v2.0 server, with scores ranging from 0.4000 to 2.000, confirming their potential as immunogenic targets. Non-allergenicity was validated using AllerTOP v2.0, while toxicity screening with ToxinPred 3.0 ensured that all shortlisted peptides were non-toxic. This rigorous screening process ensured that only safe and highly immunogenic epitopes were considered for vaccine development. The subcellular localization and membrane topology of the selected KDO transferase and TolR proteins were evaluated using PSORTb and TMHMM v2.0. TMHMM predicted a transmembrane region at residues 5–23 in KDO transferase and at residues 12–34 in TolR. The predicted B-cell epitopes were considered in relation to these topology predictions. Since computational topology does not experimentally establish surface exposure, the predicted accessibility of the B-cell epitopes should be interpreted as a computational indication rather than definitive evidence of antibody accessibility.

### 2.4. Vaccine Construction

For vaccine construction, the selected CTL, HTL, and LBL epitopes were integrated into the final design. These epitopes were connected using AAY, KK, and GPGPG linkers, chosen for their ability to enhance vaccine efficacy and epitope presentation while minimizing the risk of junctional epitope formation. The 50S ribosomal protein L7/L12, which has 124 residues, was introduced as an adjuvant and linked via the EAAAK linker, which provides structural stability, ensures effective separation of epitopes, and reduces undesired interactions with adjacent protein regions. The final vaccine construct, comprising 130 amino acids, demonstrates a strategic approach in epitope selection and linker utilization to develop an effective and robust immunization candidate.

### 2.5. Physicochemical Profiling

The designed vaccine construct underwent extensive immunogenic and physicochemical property analysis. A comparison with the human proteome confirmed no significant similarities, minimizing the risk of autoimmunity. The vaccine demonstrated high antigenicity, non-allergenicity, and non-toxicity. ProtParam analysis determined the molecular weight to be 46.28 kDa, with an isoelectric point (pI) of 7.61. Additionally, the GRAVY score was calculated as −0.065, indicating hydrophilicity. These findings collectively establish the vaccine’s suitability as a potential candidate against *L. pneumophila*.

### 2.6. Three-Dimensional Structure Prediction, Validation, Refinement, Evaluation, and Flexibility

The 3D structure of the vaccine construct was predicted using homology modeling via SWISS-MODEL. Furthermore, the top modeled structure was refined using the GalaxyRefine website. As a result, five models were generated, and model 4 was chosen based on many characteristics, including GDT-HA 0.9500, MolProbity 1.336, 96.9% Rama-favored, and an RMSD value of 0.404.

**Figure 1:**
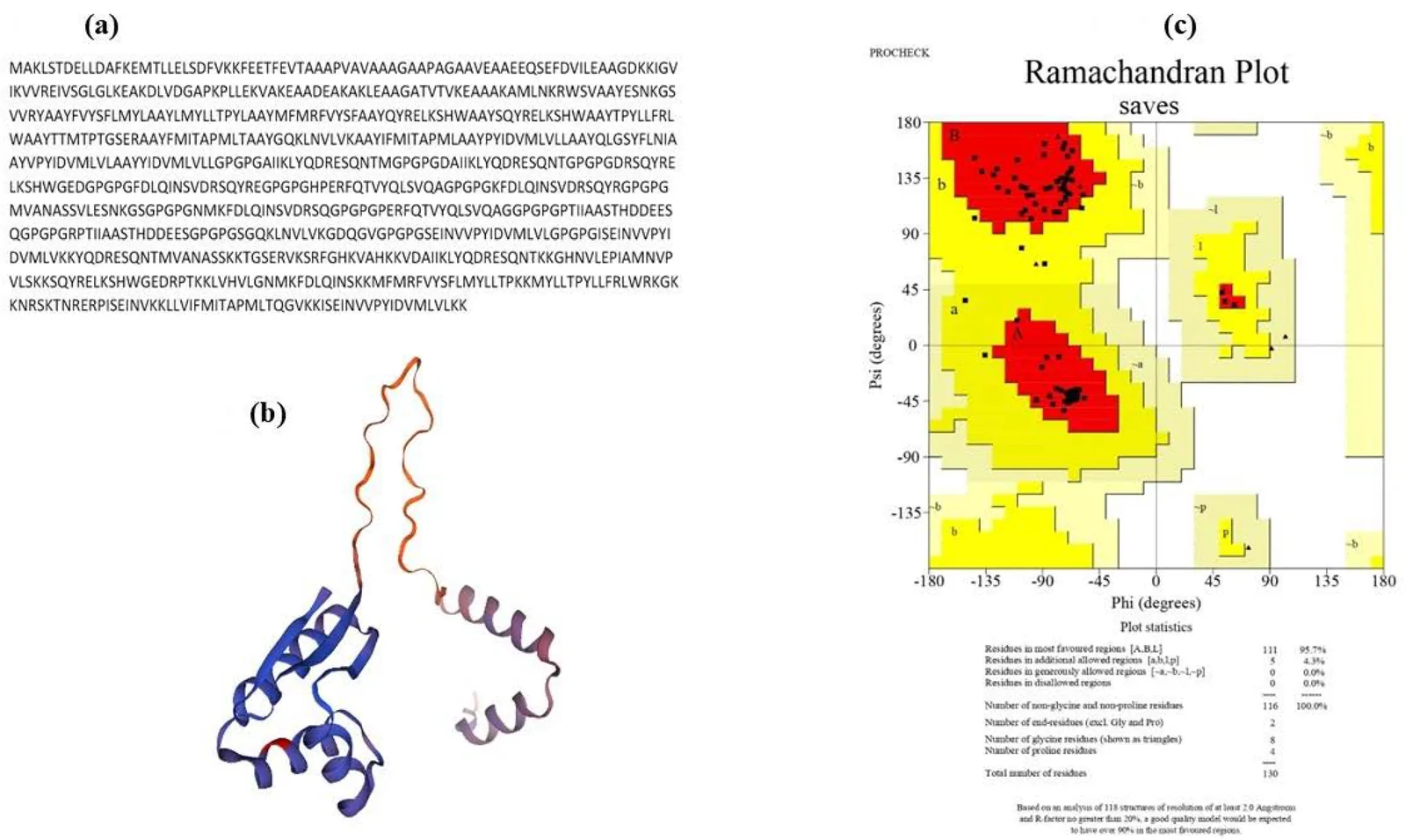
The vaccine construct sequence, along with its predicted structure and structural evaluation, is presented as follows: (**a**) displays the vaccine sequence after incorporating various linkers and an adjuvant; (**b**) illustrates the modeled vaccine structure generated using the SWISS-MODEL server; and (**c**) provides the conformational analysis, where the red zones in the Ramachandran plot represent the most favored regions, with the majority of residues positioned within these regions.

**Figure 2:**
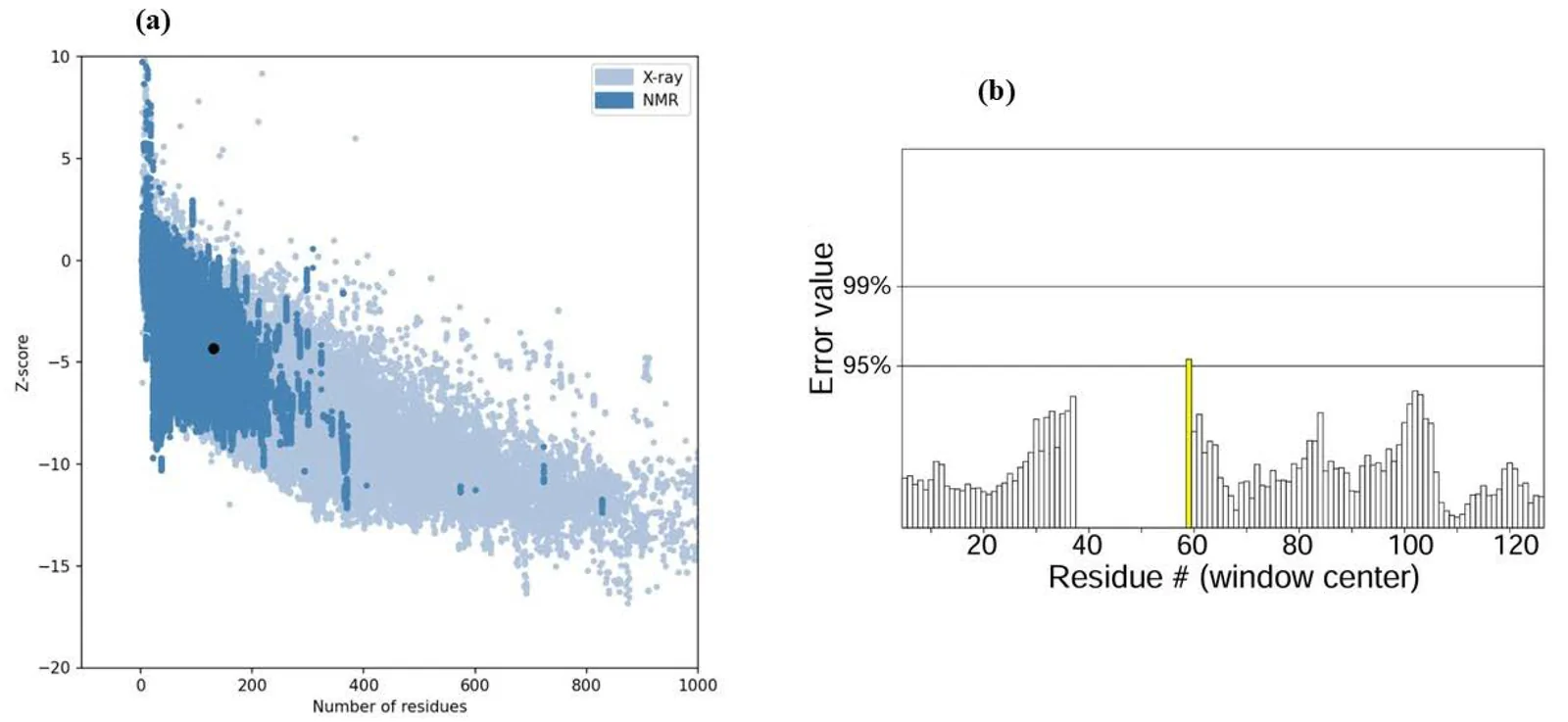
The structural quality validation. (**a**) ProSA-web Z-score analysis showing a Z-score value of −4.31. (**b**) ERRAT error prediction showing that the quality of the model is as high as 99.01%.

**Figure 3:**
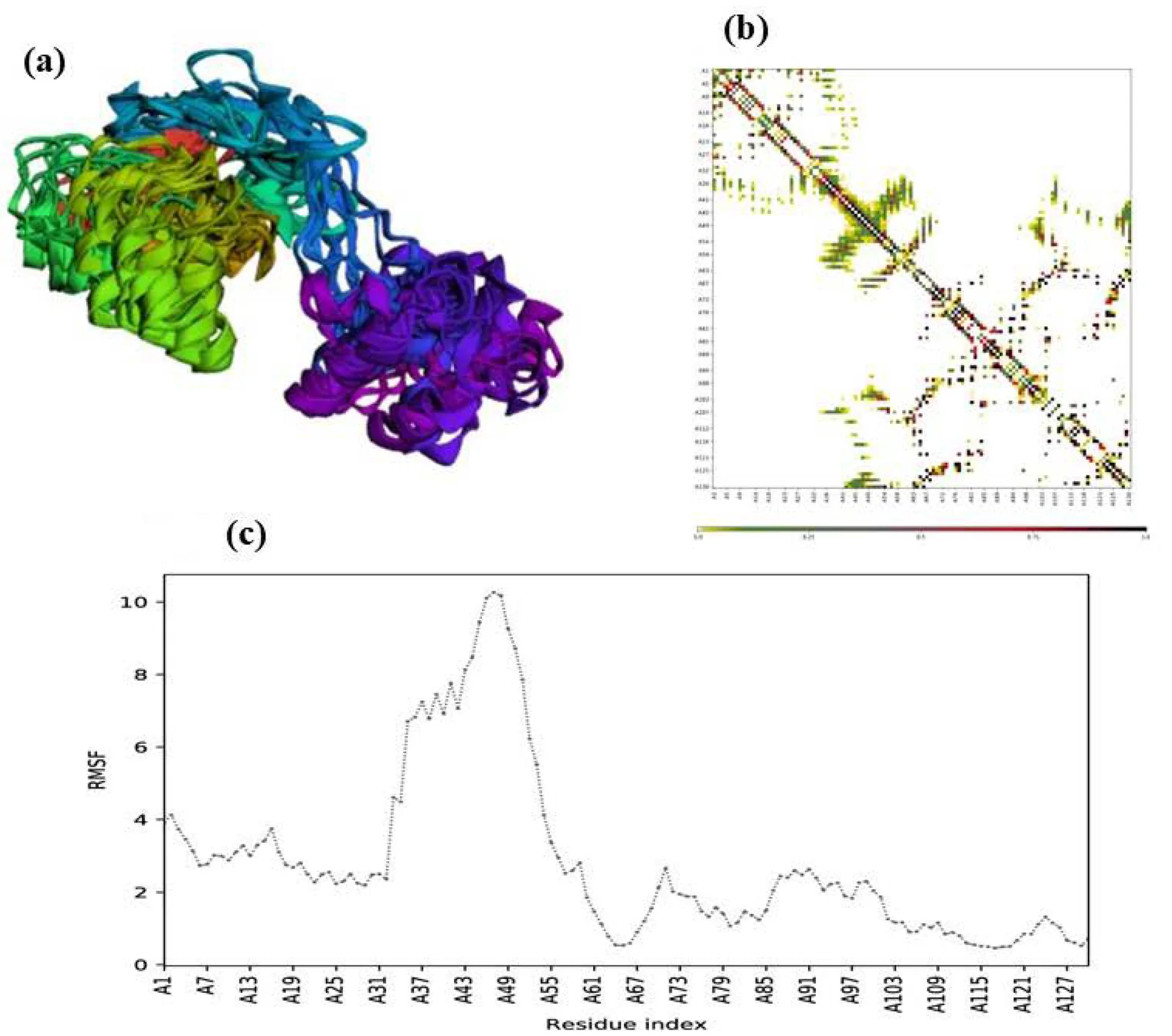
Flexibility analysis of the designed vaccine construct. (**a**) Cartoon illustration of all ten models showing minimum fluctuations, as predicted by CABS-flex 2.0. (**b**) Residue–residue interaction map. (**c**) RMSF plot showing fluctuations ranging from 0.5 Å to 10.2 Å.

According to the Ramachandran plot, 95.7% of the residues were in the most favorable regions, and 4.3% of the residues were in the additional allowed region (Figure 1). To confirm structural reliability, the refined model was further evaluated using ProSA-web and ERRAT servers for additional quality validation. ProSA-web predicted the Z-score of the protein to be −4.31. The ERRAT server was used to evaluate possible errors in the crude 3D model that might affect its overall structural integrity. The resulting quality factor of the modeled protein was 99.01%, indicating a high level of structural reliability (Figure 2). Furthermore, the flexibility of the vaccine construct was determined by an online tool, CABS-flex 2.0, which performed 50 rounds of simulation at a default temperature of 1.4 °C. The collective model of 10 retrieved structures showed fewer fluctuations near the N-terminus when compared to regions near the C-terminus (Figure 3a). The generated contact map revealed contacts among different residues of all ten final retrieved structures (Figure 3b). Finally, the fluctuation plot represented the root mean square fluctuation (RMSF) of each amino acid from 0.5 Å to 10.2 Å (Figure 3c). Such variations in the RMSF of the designed construct indicate its high flexibility, further reinforcing its potential to be used as a probable vaccine.

### 2.7. Molecular Docking of the Designed Vaccine Candidate

**Figure 4:**
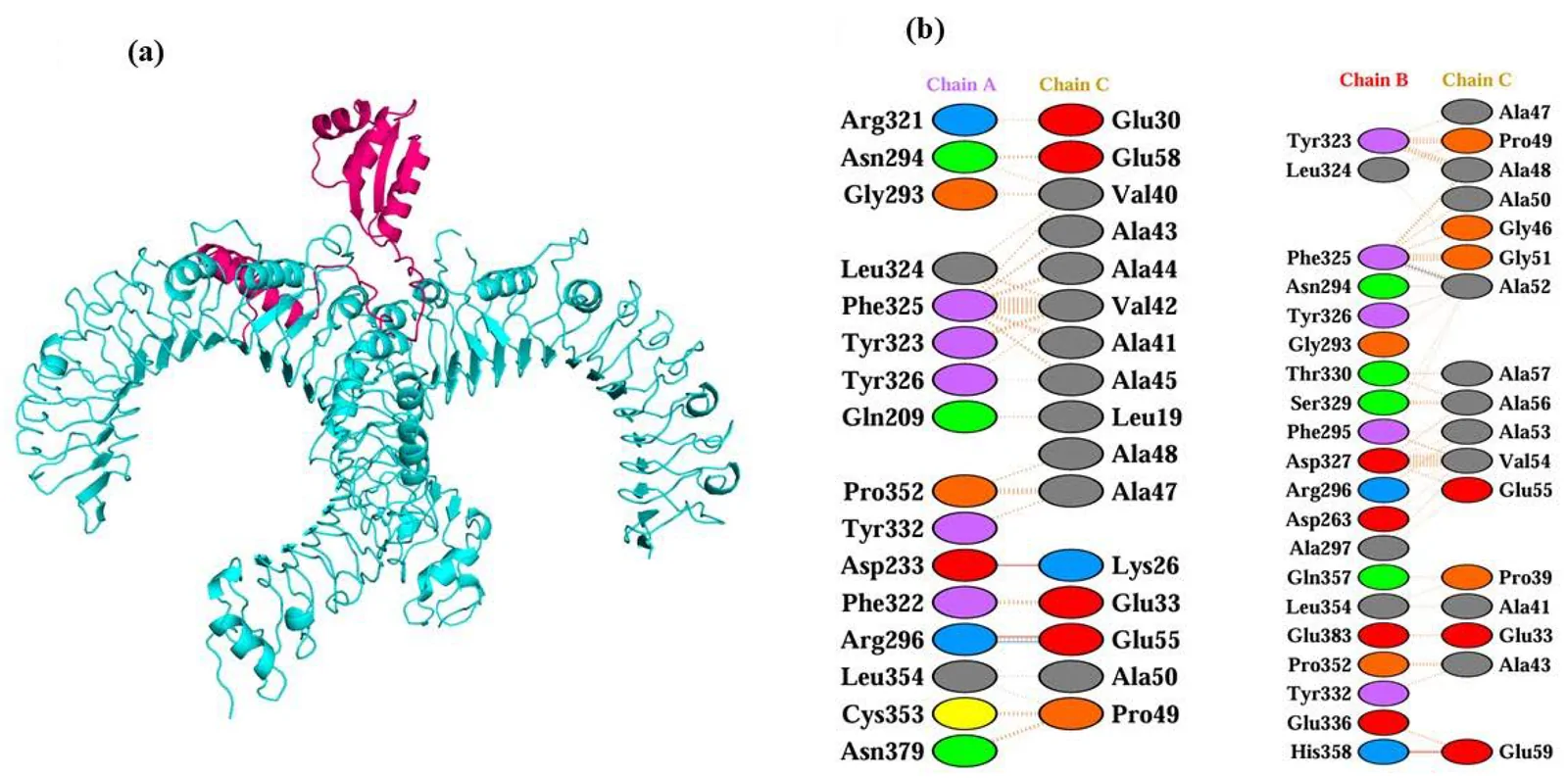
Molecular docking with TLR-2 and interaction analysis. (**a**) Cartoon representation of the docked complex of TLR-2 (cyan blue) and the designed vaccine construct (hot pink). (**b**) Interacting residues between the two chains (A and B) of TLR-2 and the designed vaccine construct chain (C).

**Figure 5:**
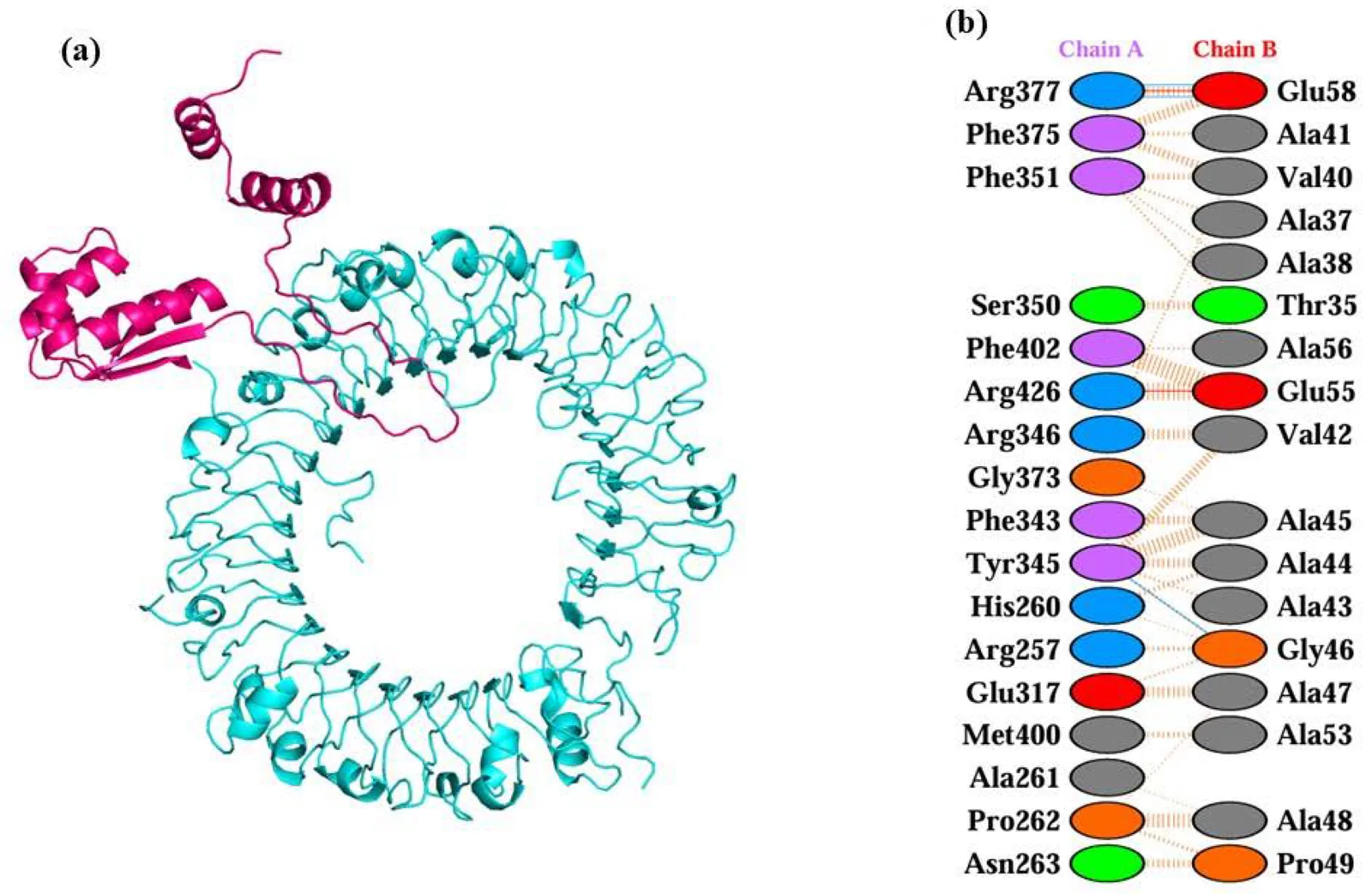
Molecular docking with TLR-9 and interaction analysis. (**a**) Cartoon representation of the docked complex of TLR-9 (cyan blue) and the designed vaccine construct (hot pink). (**b**) Interacting residues between chain (A) of TLR-9 and the designed vaccine construct chain (C).

Molecular docking of the designed vaccine construct was carried out against TLR-2 (PDB ID: 6NIG) and TLR-9 (PDB ID: 3WPF) using BioLuminate protein–protein docking. As a result of docking, the top-ranked model was considered based on a greater PIPER cluster size. The top-ranked models, TLR-2 and TLR-9, when complexed with the vaccine construct, generated a PIPER cluster size of 84 and 126, respectively (Figure 4 and Figure 5). The complexes were analyzed to determine binding affinity and interaction stability. Key hydrogen bond interactions were identified, and further docking data revealed substantial interactions within the receptor–protein complex. The PDBsum analysis of the docking complexes provided detailed structural information, including residue interactions categorized based on their physicochemical properties. Residues were classified as positive (red), negative (blue), neutral (green), aliphatic (yellow), aromatic (purple), and cysteine (orange), providing details on their electrostatic and hydrophobic nature. Overall, two hydrogen bonds and three salt bridges were observed between the vaccine construct and TLR-2. In the case of TLR-9, three hydrogen bonds and two salt bridges were identified, thus contributing to the stability of the complexes.

### 2.8. Molecular Dynamic Simulation Analysis

**Figure 6:**
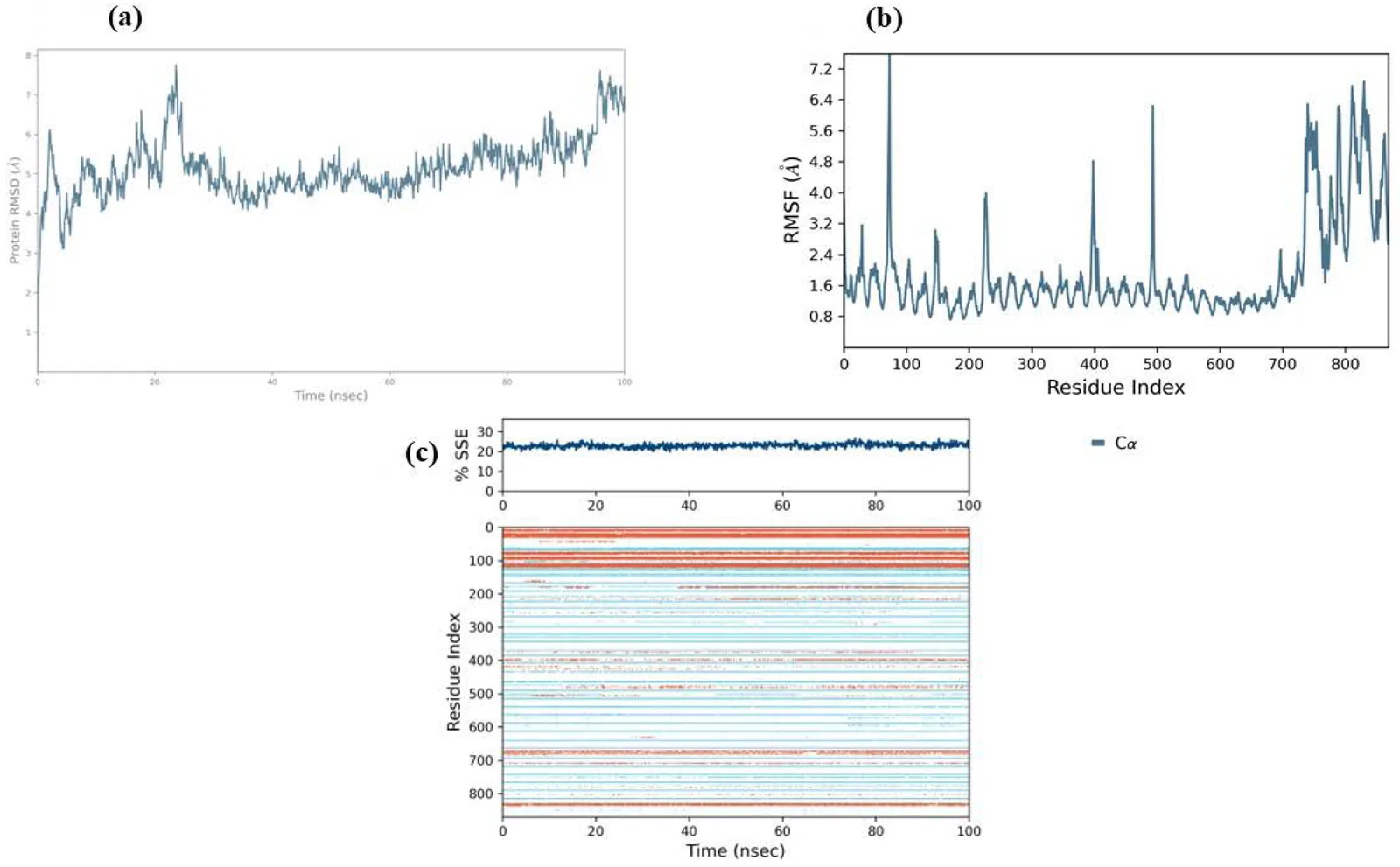
Molecular dynamics simulation analysis of the TLR-9–vaccine complex. (**a**) The RMSD plot of the TLR-9–vaccine complex. (**b**) The RMSF plot of the TLR-9–vaccine complex. (**c**) TLR-9–vaccine secondary structure changes during simulation, where light blue refers to β-strands and orange refers to α-helices.

The 100 ns molecular dynamics simulation of the TLR-9–vaccine complex showed that the interaction between the two is stable along the trajectory. The protein backbone RMSD value stabilized following initial equilibration and hence indicated no major fluctuations occurring during the production run (Figure 6a). Furthermore, the RMSD fluctuations were within the range usually seen for large protein complexes and hence suggested that this vaccine construct is structurally compatible with the TLR-9 binding interface. The RMSF analysis showed limited fluctuation at the residue level, with flexibility mainly confined to terminal residues and exposed loop regions. The core interacting residues had low RMSF values, with stable binding of the vaccine construct to the TLR-9 binding pocket confirmed (Figure 6b). The secondary structure analysis indicated that throughout the simulation, the protein did not lose its structural integrity, with ~10.08% helix and ~12.91% strand content, which indicated that it neither unfolded nor collapsed. This is further supported by a consistent profile of secondary structures (Figure 6c). Overall, the TLR-9–vaccine complex remained conformationally stable, presenting favorable and sustained interactions along the 100 ns simulation period, supporting the reliability of the vaccine–receptor complex.

**Figure 7:**
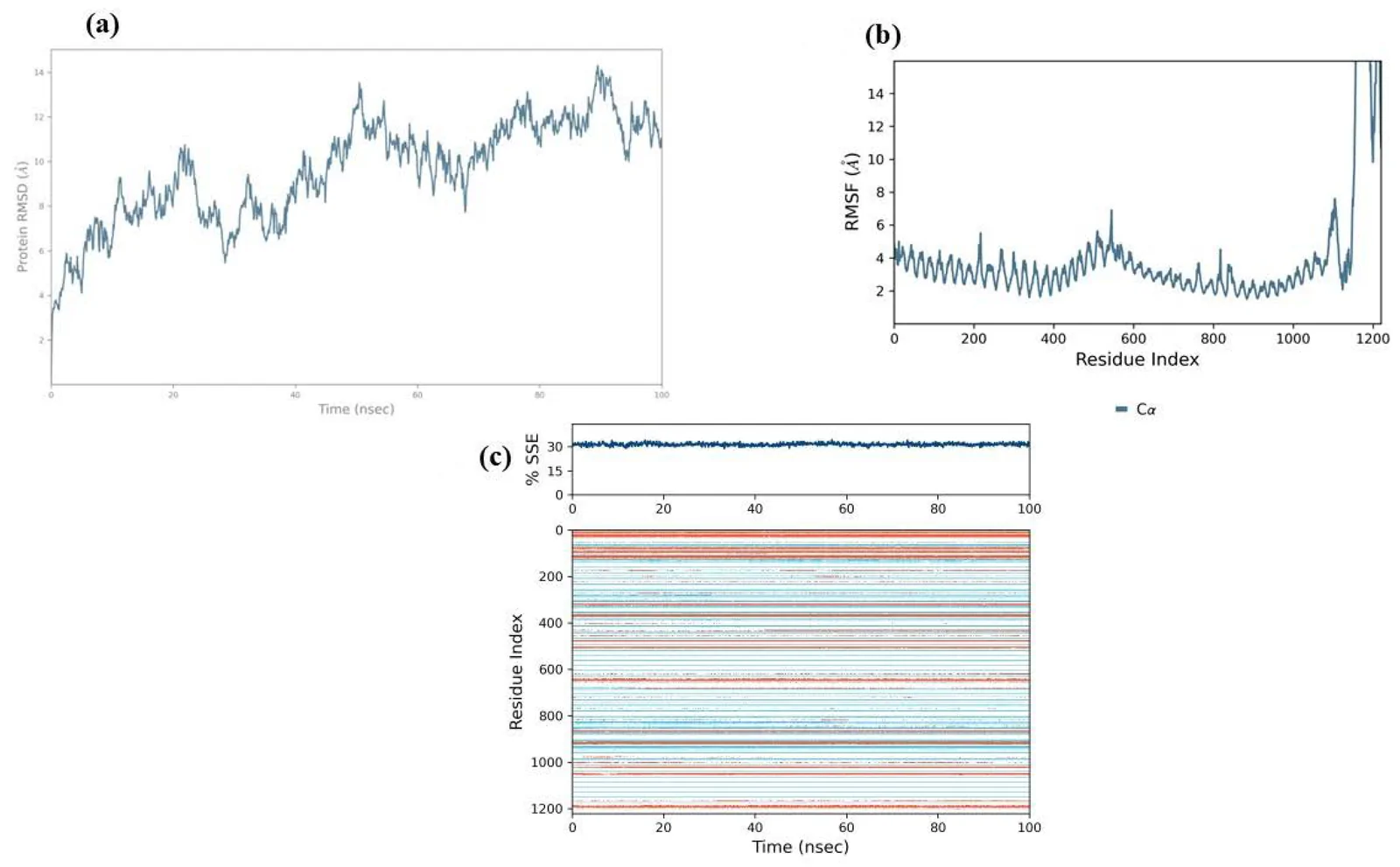
Molecular dynamics simulation analysis of the TLR-2–vaccine complex. (**a**) The RMSD plot of the TLR-2–vaccine complex. (**b**) The RMSF plot of the TLR2–vaccine complex. (**c**) TLR-2–vaccine secondary structure changes during simulation, where light blue refers to β-strands and orange refers to α-helices.

The TLR-2–vaccine complex exhibited good stability during the entire course of the 100 ns simulation. After an initial fluctuation, the backbone RMSD reached an equilibrium value and remained stable for the rest of the trajectory (Figure 7a). Analysis of the RMSF profile indicated that most fluctuations occurred at flexible loop regions and terminal segments, while the residues at the vaccine interaction interface showed minimal deviations, confirming stable binding and limited local perturbations (Figure 7b). Secondary structure assessment showed TLR-2 to have maintained a well-defined fold throughout the course of the simulation, with ~13.86% helix and ~17.47% β-strand content. No significant loss in structural elements was observed, confirming that vaccine binding does not disrupt the stability of the overall receptor complex (Figure 7c). Collectively, the RMSD, RMSF, and secondary structure evaluations indicate that the TLR-2–vaccine complex is dynamically stable, with strong and consistent molecular interactions maintained during the simulation.

### 2.9. Codon Optimization and Cloning

**Figure 8:**
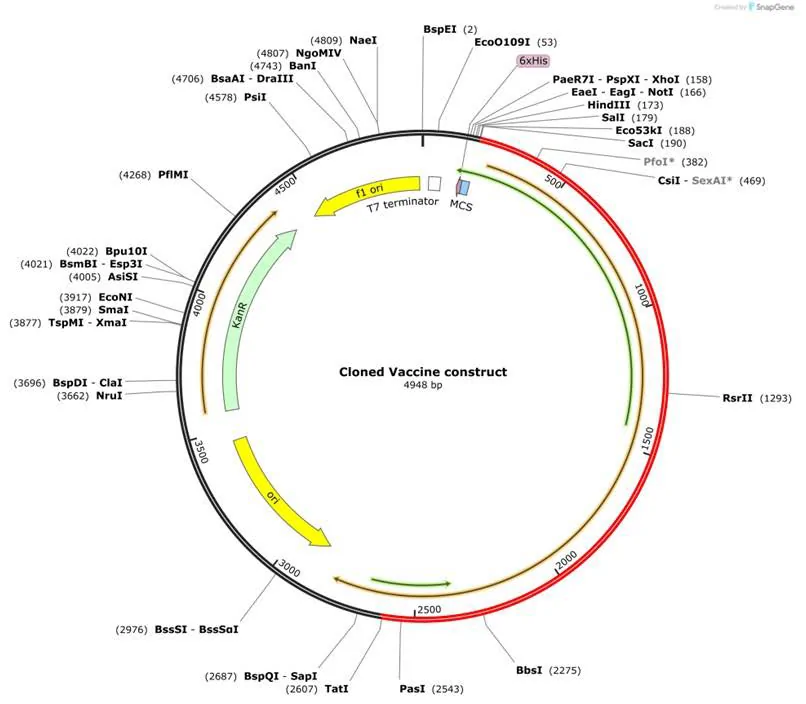
Cloned vaccine construct (red color) in the PET28a (+) vector (black backbone) after codon optimization.

Codon optimization was performed, followed by in silico cloning of the designed vaccine construct into the *E. coli* expression vector. The codons of the vaccine construct were optimized to match those preferred by the *E. coli* system using the JCat web server. The resulting cDNA sequence comprised 2724 nucleotides. A CAI value of 0.96 was observed, and the GC content (50.18%) was within the optimal range for efficient protein expression. The restriction enzymes Eco53kI and TatI were used to digest the optimized construct’s 5′ and 3′ ends, respectively. After the digested fragment was successfully ligated into the pET28a (+) vector, a recombinant plasmid with roughly 4948 base pairs was created. SnapGene software 8.2.3 was used to visualize the recombinant vector map (Figure 8).

### 2.10. Analysis of Immune Simulation

**Figure 9:**
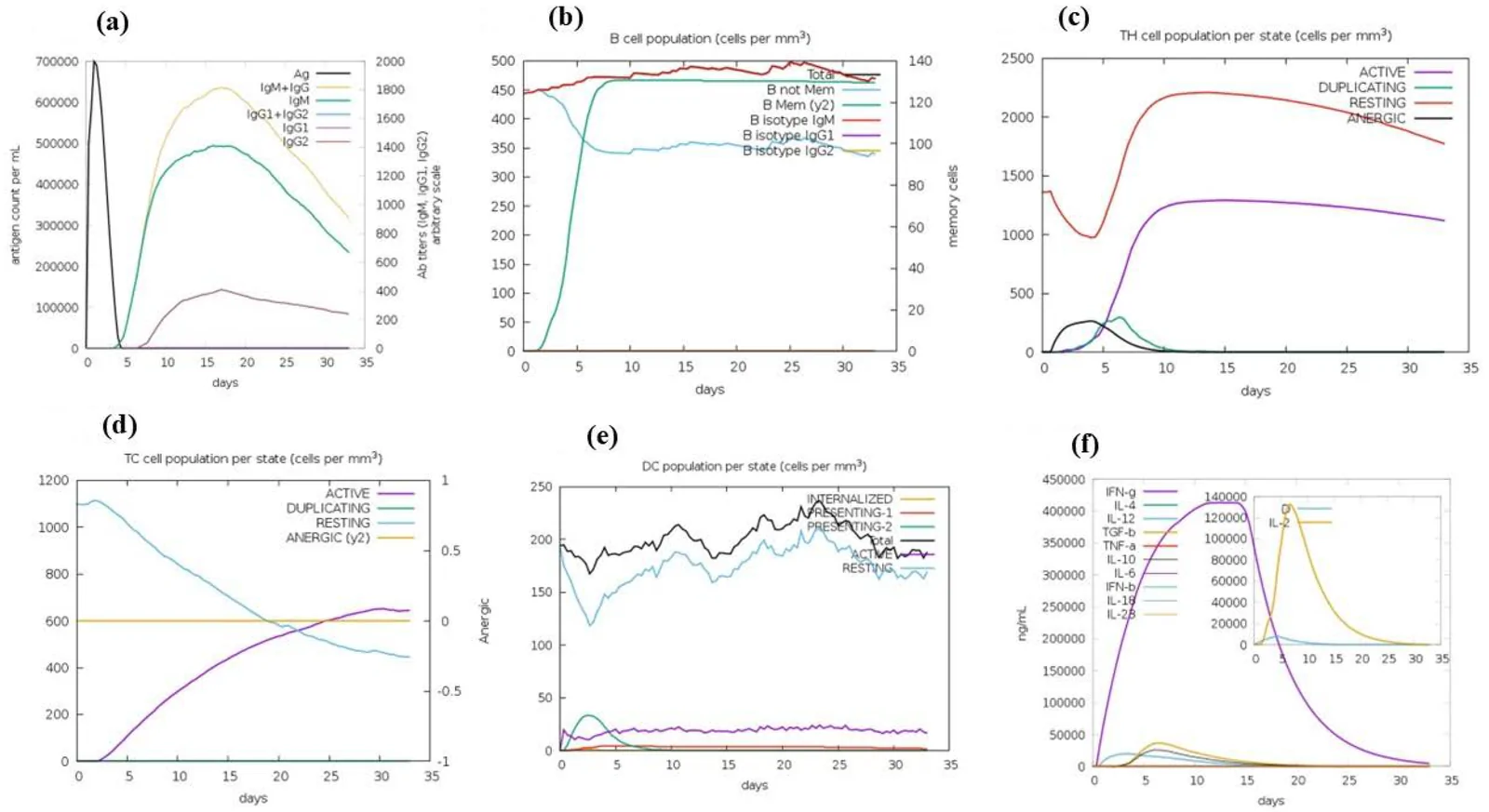
Illustration of immune simulation properties of the designed vaccine construct, as assessed by C-ImmSim. (**a**) Immunoglobulins and immune complex graph. (**b**) Number of B-cell types in immune responses. (**c**) Helper T-cell populations. (**d**) Cytotoxic T-cell populations. (**e**) Dendritic cells per state. (**f**) Response graph generated for cytokines and interleukins in the designed vaccine.

The simulation predicted fast antigen clearance within 5 days, and a reasonable spike in antibody levels was observed (Figure 9a). B-cell dynamics showed early and then sustained expansion of memory B cells, with a corresponding reduction in non-memory B cells, consistent with durable humoral memory (Figure 9b). Dendritic cells had an early activated phase during days 1 to 4, and then stabilized as the antigen was cleared (Figure 9e). The cytokine milieu is Th1-skewed, with an early IL-2 burst supporting clonal expansion and a pronounced IFN-γ peak at around day 15, while IL-4/TGF-β remained low (Figure 9f). In addition, both CD8^+^ cytotoxic T cells (Figure 9d) and CD4^+^ helper T cells (Figure 9c) expanded, followed by partial contraction with negligible anergy, indicating effective priming and memory rather than tolerization. Collectively, these in silico results seem to indicate that a single dose of this vaccine candidate may induce coordinated humoral and cell-mediated responses with a favorable Th1 profile.

## 3. Discussion

The proteome era has revolutionized pathogen research, offering vast genomic and proteomic data to identify new therapeutic targets and improve personalized medicine, driven by advancements in sequencing technologies [21,22]. Computational methods have accelerated vaccine development, with vaccine informatics enabling in silico design and the prediction of immunogenic epitopes. This approach enhances vaccine efficacy by targeting both cellular and humoral immune responses. Additionally, understanding pathogen proteins through genomic and proteomic data is crucial for designing vaccines that effectively target key proteins involved in disease progression. Previous studies emphasize the importance of stimulating both cellular and humoral immunity for effective vaccine development [23,24].

In the present study, we employed the complete proteome of *Legionella pneumophila* subsp. *pneumophila* strain L01C.1 (Proteome ID: UP000277145) as the basis for a comprehensive proteome-wide reverse vaccinology analysis. Systematic screening enabled the identification of essential, virulence-associated, surface-accessible, and antigenic proteins suitable for multi-epitope vaccine design. Among the shortlisted candidates, KDO transferase and TolR were selected based on their essential biological functions, antigenicity, and lack of significant homology to human proteins. Although the computational analyses were performed using a single strain-specific proteome, both proteins participate in fundamental bacterial processes associated with lipopolysaccharide biosynthesis and outer membrane integrity, supporting their potential as promising vaccine targets. Nevertheless, comparative analyses involving additional *L. pneumophila* strains, together with experimental validation, will be important to determine the breadth of protection offered by the proposed vaccine.

In this study, we implemented a systematic bioinformatics approach to design a multi-epitope vaccine candidate targeting *Legionella pneumophila*, the causative agent of Legionnaires’ disease. The methodology encompassed multiple steps, including the retrieval of the bacterial proteome, identification of essential and immunogenic proteins, epitope prediction, vaccine construction, molecular docking analysis, molecular dynamics simulation, immune simulation, codon optimization, and cloning. Our primary objective was to identify optimal vaccine candidates capable of stimulating both humoral and cellular immune responses while minimizing potential adverse effects, such as allergenicity and toxicity. The findings suggest that the bioinformatics-driven multi-epitope vaccine design presents a promising therapeutic strategy against *L. pneumophila* infections. Two candidate proteins were selected for further analysis: 3-deoxy-D-manno-octulosonic acid transferase and the Tol-Pal system protein TolR. These proteins were found to have high antigenicity scores and no significant homology with human proteins, which minimizes the risk of autoimmunity. Among the shortlisted proteins, KDO transferase and TolR were prioritized through the convergence of multiple criteria, including predicted essentiality, virulence association, cellular localization, antigenicity, and the absence of significant similarity to human proteins. Their selection was therefore based on both biological relevance to bacterial survival and pathogenicity and their predicted suitability for eliciting a specific host immune response.

3-deoxy-D-manno-octulosonic acid (KDO) transferase (KDO transferase) is essential for the biosynthesis of lipopolysaccharides (LPS), which are crucial components of the outer membrane in Gram-negative bacteria. KDO transferase facilitates the transfer of KDO to the lipid A-core oligosaccharide precursor, ensuring the structural integrity and functionality of LPS, which are known virulence factors that aid in immune evasion, host cell adherence, and biofilm formation [25,26]. Disruption of KDO transferase has been shown to compromise bacterial viability and reduce virulence, making it a promising target for vaccine development. The antigenicity analysis of KDO transferase indicates high potential for stimulating an immune response, while its lack of homology with human proteins reduces the risk of autoimmune reactions, enhancing its safety as a vaccine candidate.

The Tol-Pal system protein TolR is also significant in bacterial pathogenesis. It plays a key role in maintaining outer membrane stability and facilitating cell division in Gram-negative bacteria. This system is particularly important for the survival and infectivity of *Legionella pneumophila*, as it helps maintain outer membrane integrity under the stress conditions encountered during host infection [27]. TolR contributes to the energy transduction necessary for the proper functioning of the Tol-Pal system. Its role in bacterial pathogenesis makes it an attractive target for immunological interventions. Antigenicity and epitope predictions for TolR further support its potential as a vaccine candidate, with several epitopes identified that could effectively activate both B cell- and T cell-mediated immune responses [25,27].

After identifying the target proteins, B-cell and T-cell epitopes were predicted using a combination of well-established computational tools such as ABCpred and IEDB. The selected epitopes were evaluated based on their binding affinity to major histocompatibility complex (MHC) molecules and their immunogenic potential. This approach ensured that the epitopes could effectively induce both humoral and cellular immune responses while minimizing the risk of adverse reactions. To further enhance immunogenicity, a multi-epitope vaccine was constructed by linking the selected B-cell and T-cell epitopes using appropriate linkers, which were specifically chosen to preserve the structural integrity and functionality of each epitope. This study utilized advanced bioinformatics tools to predict T-cell and B-cell epitopes, identifying immunogenic sequences suitable for inclusion in a multi-epitope vaccine.

The predicted 3D model showed that it was 96.9% Rama-favored, and produced a Z-score and ERRAT value of −4.31 and 99.01%, respectively. The Z-score’s consistency with the X-ray-derived structure supports the reliability of the proposed model. According to the study conducted by Fuse et al., it is evident that TLR-2 plays an important role in the recognition of *L. pneumophila*. TLR-9 has likewise been reported to contribute to the host immune response against *L. pneumophila* [28,29]. Therefore, based on these findings, we performed molecular docking of the designed vaccine construct with TLR-2 and TLR-9. We then adapted the sequence to *E. coli* codon usage (compatible with pET-28a (+)), enhancing protein yield and purification. The human immune reaction was modeled with C-ImmSim in silico to characterize predicted humoral and cellular dynamics. Molecular docking analyses revealed strong interactions between the vaccine construct and human toll-like receptors (TLR-2 and TLR-9). The strong binding affinity and stability of these complexes further enhance their potential as vaccine targets. In addition, interactions between the designed vaccine and TLR-2 and TLR-9 were evaluated using the docking process, and the docking complexes were stabilized using MD simulations.

This research contributes to the growing field of vaccine development for *Legionella pneumophila*, proposing a novel approach that could stimulate both cellular and humoral immunity. However, to establish the safety and efficacy of the proposed vaccine, experimental validation through in vitro and in vivo studies is essential. These validations will be crucial in advancing vaccine development and contributing to future strategies for preventing Legionnaires’ disease. A limitation of the present study is that the reverse vaccinology pipeline was performed using a single *L. pneumophila* proteome. A limitation of this study is that the vaccine target identification was based on the L01C.1 strain and therefore does not establish conservation of the selected proteins and epitopes across diverse *L. pneumophila* isolates. Comparative analysis using multiple strains or pan-genome datasets will be required to determine their broader applicability. Future comparative analyses incorporating the reference proteome together with additional clinical isolates or pan-genome datasets will facilitate the identification of conserved vaccine targets and universally conserved epitopes, thereby improving the potential breadth of strain coverage. Furthermore, experimental validation is required to confirm the immunogenicity, safety, and protective efficacy of the proposed vaccine construct.

## 4. Materials and Methods

### 4.1. Proteome Retrieval and Identification of Essential Proteins

The complete proteome of *Legionella pneumophila* subsp. *pneumophila* strain L01C.1 (Proteome ID: UP000277145) was retrieved from the UniProt database in FASTA format. The dataset comprises 3218 annotated protein sequences and served as the basis for the present proteome-wide reverse vaccinology analysis. Essential proteins were identified using the Geptop 2.0 server, which predicts indispensable genes based on orthology and phylogenetic profiles [30,31].

Subcellular localization analysis was performed using PSORTb to identify outer-membrane proteins, as these are considered prime vaccine targets. Virulence-associated proteins were screened using VIRULENTPRED [32]. Transmembrane helices were predicted using TMHMM v2.0 [33], and physicochemical properties, including molecular weight, were calculated using ExPASy ProtParam [34].

### 4.2. Selection of Immunogenic Proteins

To identify potential vaccine candidates, the antigenicity of the shortlisted proteins was evaluated using VaxiJen v2.0 with a threshold of 0.5, allowing the selection of proteins with a high probability of eliciting an immune response [35]. To ensure safety and reduce the risk of autoimmune cross-reactivity, the selected antigenic proteins were further screened for similarity against the human proteome using BLASTp. Proteins showing significant homology were excluded from further analysis.

### 4.3. Epitope Prediction and Screening

B-cell and T-cell epitopes were predicted from the selected antigenic proteins. Linear B-cell epitopes were identified using ABCpred with an epitope length of 14 amino acids [36]. T-cell epitopes, including both MHC Class I and Class II binders, were predicted using the IEDB resource, selecting peptides with a percentile rank ≤ 1.0 to ensure high binding affinity [37]. Predicted T-cell epitopes ranged between 9–11 amino acids in length. All shortlisted epitopes were further evaluated for antigenicity using VaxiJen v2.0, allergenicity using AllerTOP v2.0, and toxicity using ToxinPred. Only epitopes that were antigenic, non-allergenic, and non-toxic were selected for subsequent analysis [38].

### 4.4. Multi-Epitope Vaccine Construction

A multi-epitope vaccine construct was designed by assembling cytotoxic T lymphocyte (CTL), helper T lymphocyte (HTL), and linear B lymphocyte (LBL) epitopes. Appropriate linkers were incorporated to maintain structural integrity and enhance epitope presentation: AAY linkers for CTL epitopes, GPGPG for HTL epitopes, and KK linkers for B-cell epitopes. To enhance immunogenicity, the 50S ribosomal protein L7/L12 was included as an adjuvant. Additionally, the synthetic peptide RS-09 (APPHALS), a known TLR4 agonist, was linked using an EAAAK linker to improve immune activation. The inclusion of adjuvants and linkers was aimed at improving the stability, folding, and immunogenic efficiency of the final construct [39].

### 4.5. Physicochemical and Structural Characterization

The final vaccine construct was analyzed for similarity against the human proteome using BLASTp 2.17.0 to confirm non-homology. Physicochemical properties, including molecular weight, theoretical isoelectric point (pI), extinction coefficient, and half-life, were computed using ExPASy ProtParam. Antigenicity and allergenicity were re-evaluated using VaxiJen v2.0 and AllerTOP v2.0, respectively. Secondary structure elements, including α-helices, β-sheets, turns, and random coils, were predicted using SOPMA [40,41]. Solubility upon expression was assessed using SOLpro 2.0 [42,43].

### 4.6. 3D Structure Modeling, Refinement, and Validation

The three-dimensional structure of the vaccine construct was generated using homology modeling via SWISS-MODEL. Model quality was evaluated using QMEANDisCo scoring. Structural refinement was performed using the GalaxyRefine server [44]. The refined model was validated using PROCHECK for Ramachandran plot analysis, ProSA-web for Z-score evaluation, and ERRAT for identifying structural anomalies. Structural flexibility was further assessed using CABS-Flex 2.0 [45].

### 4.7. Molecular Docking Analysis

Molecular docking was performed to evaluate the interaction between the vaccine construct and immune receptors, specifically Toll-like receptor 2 (TLR-2) and Toll-like receptor 9 (TLR-9), which are known to play roles in immune responses against Gram-negative pathogens [28,29]. Docking was conducted using BioLuminate 2.7. Both receptor and ligand structures were prepared using the Protein Preparation Wizard with the OPLS3 force field. The docking protocol included 70,000 ligand rotations and a maximum of 30 poses per ligand. The active site residues of the TLR2 and cocrystal ligand (diprovocim) were identified using PyMOL 3.1.8. The active site residues were chain a: phe349, leu350, pro352, glu375, tyr376, and asn379, and chain b: ile261, leu266, met270, leu273, leu282, phe284, cys287, the288, leu289, pro306, val309, leu312, ile314, leu317, ile319, tyr323, phe325, tyr326, leu328, leu331, tyr332, leu334, thr335, val338, ile341, val343, ser346, lys347, phe349, leu350, val351, pro352, leu355, leu359, leu365, and leu367. For the TLR-9 protein (PDB ID: 3WPF), the blind docking strategy was performed due to the unavailability of the cocrystal ligand. Interaction stability and residue-level contacts were analyzed using PDBsum to characterize binding interfaces and interaction patterns.

### 4.8. Molecular Dynamics Simulation

To evaluate the stability of the docked complexes, molecular dynamics (MD) simulations were performed using Desmond for 100 ns. The system was solvated using a TIP3P water model within an orthorhombic box, maintaining a buffer distance of 10 Å. Physiological conditions were simulated by adding 0.15 M NaCl. Energy minimization was conducted for 100 ps prior to the production run. Simulations were performed under an NPT ensemble at a temperature of 300 K and a bar pressure of 1.01325. Temperature and pressure were maintained using the Nosé–Hoover thermostat and Martyna–Tobias–Klein barostat, respectively. Trajectory data were recorded at intervals of 1.2 ps.

### 4.9. Codon Optimization and In Silico Cloning

Codon optimization of the designed vaccine construct was performed using JCat to enhance translational efficiency in Escherichia coli K12, a commonly used expression host [46,47]. The optimization process aimed to improve the codon adaptation index (CAI), GC content, and overall expression potential while avoiding rho-independent transcription terminators, prokaryotic ribosome-binding sites, and restriction enzyme cleavage sites. Following optimization, restriction sites for Eco53kI and TatI were introduced at the N- and C-termini, respectively, to facilitate directional cloning. The optimized gene sequence was then inserted into the pET28a (+) expression vector using SnapGene software (version 8.2.1), enabling in silico validation of cloning strategy and construct integrity.

### 4.10. Immune Simulation

The immunogenic profile of the designed vaccine was evaluated using C-ImmSim, an agent-based simulation platform that models mammalian immune responses. This tool utilizes a position-specific scoring matrix (PSSM) along with machine learning algorithms to predict immune interactions over time. The simulation was performed using a random seed of 12345, a simulation volume of 10, and 100 simulation steps. The HLA settings were: both MHC Class I A alleles are HLA-A*01:01, both MHC class I B alleles are HLA-B*07:02, and both MHC class II DR alleles are HLA-DRB1*01:01. The vaccine is used without any LPS in which the adjuvant value is 100, and the antigen value is 1000. The first dose of the vaccine is delivered at time step 1. No replicates were performed.

The vaccine sequence was used as input to simulate primary and secondary immune responses, including antigen presentation, B-cell and T-cell activation, memory cell formation, and cytokine production. Multiple time-step simulations were conducted to assess the consistency and strength of the immune response, thereby providing insights into the potential efficacy and immunogenicity of the vaccine construct [48].

## 5. Conclusions

In this study, a novel multi-epitope vaccine candidate targeting *Legionella pneumophila* was rationally designed using integrated immunoinformatics and reverse vaccinology approaches. Key antigenic proteins, including KDO transferase and TolR, were identified as promising targets based on their essentiality, antigenicity, and non-homology to the human proteome. The selected epitopes demonstrated favorable immunological properties, including high antigenicity, non-allergenicity, and non-toxicity, supporting their suitability for vaccine development. The constructed multi-epitope vaccine exhibited desirable physicochemical characteristics and structural stability, while molecular docking analysis revealed strong binding affinity with immune receptors TLR-2 and TLR-9, suggesting its potential to effectively stimulate innate and adaptive immune responses. Furthermore, immune simulation results indicated the capability of the vaccine construct to induce robust humoral and cellular immunity, including memory response generation. Given the absence of a licensed vaccine against *L. pneumophila*, the present study provides a promising computational framework for vaccine design. However, experimental validation through in vitro and in vivo studies remains essential to confirm the immunogenicity, safety, and protective efficacy of the proposed vaccine construct. This study is based exclusively on computational analyses; therefore, the predicted antigenicity, immunogenicity, receptor interactions, and structural stability cannot be considered experimental evidence of vaccine efficacy. The predicted interactions with TLR2 and TLR9 and the immune responses require experimental validation to confirm receptor activation and immunogenicity. In addition, the conservation of the selected targets and epitopes across diverse *L. pneumophila* strains remains to be established. Further in vitro and in vivo studies are required to evaluate the safety, immunogenicity, and protective efficacy of the proposed vaccine candidate. These findings lay a strong foundation for the future development of effective prophylactic strategies against Legionnaires’ disease.

## Figures and Tables

**Table 1:** Vaccine candidates.

Protein ID	Protein Name	Gene Name	Organism
A0A3A6UMR 5	3-deoxy-D-manno-octulosonic acid transferase	D1H98_08590	*Legionella pneumophila* subsp. *Pneumophila*
A0A3A6UW18	Tol-Pal system protein TolR	tolR	*Legionella pneumophila* subsp. *Pneumophila*

**Table 2:** Epitopes predicted by ABCpred.

List of B-Cell Epitopes Predicted by ABCpred					
**UMR5 BCELL**					
**Rank**	**Sequence**	**Start Position**	**Score**	**Allergenicity**	**Antigenicity**
3	YQDRESQNTMVANASS	384	0.91	Non-Allergen	ANTIGEN
5	TGSERVKSRFGHKVAH	87	0.89	Non-Allergen	ANTIGEN
7	VDAIIKLYQDRESQNT	377	0.83	Non-Allergen	ANTIGEN
8	GHNVLEPIAMNVPVLS	332	0.82	Non-Allergen	ANTIGEN
11	SQYRELKSHWGEDRPT	218	0.73	Non-Allergen	ANTIGEN
12	LVHVLGNMKFDLQINS	199	0.72	Non-Allergen	ANTIGEN
14	MFMRFVYSFLMYLLTP	1	0.7	Non-Allergen	ANTIGEN
21	MYLLTPYLLFRLWRKG	11	0.6	Non-Allergen	ANTIGEN
**UM18 BCEL** **L**					
**Rank**	**Sequence**	**Start position**	**Score**	**Allergenicity**	**Antigenicity**
4	NRSKTNRERPISEINV	2	0.75	Non-Allergen	ANTIGEN
5	LLVIFMITAPMLTQGV	27	0.72	Non-Allergen	ANTIGEN
7	ISEINVVPYIDVMLVL	12	0.62	Non-Allergen	ANTIGEN

**Table 3:** List of IEDB-tool-generated peptides for MHC 1.

UMR5 MHC1					
Allele	Peptide	Percentile Rank	Antigenicity	Toxicity	Allergenicity
[‘HLA-A*02:01’, ‘HLA-B*08:01’, ‘HLA-A*02:03’, ‘HLA-A*02:06’, ‘HLA-A*32:01’]	AMLNKRWSV	0.08	ANTIGEN	Non-Toxin	Non-Allergen
[‘HLA-B*08:01’, ‘HLA-B*51:01’]	DAMLNKRWSV	0.56	ANTIGEN	Non-Toxin	Non-Allergen
[‘HLA-A*33:01’]	DLQINSVDR	0.89	ANTIGEN	Non-Toxin	Non-Allergen
[‘HLA-B*53:01’, ‘HLA-B*35:01’, ‘HLA-B*51:01’]	EPIAMNVPVL	0.62	ANTIGEN	Non-Toxin	Non-Allergen
[‘HLA-A*68:01’, ‘HLA-A*33:01’]	ESNKGSVVR	0.06	ANTIGEN	Non-Toxin	Non-Allergen
[‘HLA-A*26:01’, ‘HLA-A*01:01’, ‘HLA-A*30:02’, ‘HLA-B*35:01’]	ESNKGSVVRY	0.07	ANTIGEN	Non-Toxin	Non-Allergen
[‘HLA-A*68:02’]	ETELWPNLI	0.73	ANTIGEN	Non-Toxin	Non-Allergen
[‘HLA-B*15:01’, ‘HLA-A*26:01’, ‘HLA-B*35:01’]	FLMYLLTPY	0.43	ANTIGEN	Non-Toxin	Non-Allergen
[‘HLA-A*02:01’, ‘HLA-A*02:03’]	FLMYLLTPYL	0.42	ANTIGEN	Non-Toxin	Non-Allergen
[‘HLA-B*08:01’]	FMRFVYSFL	0.72	ANTIGEN	Non-Toxin	Non-Allergen
[‘HLA-A*02:06’, ‘HLA-A*02:03’, ‘HLA-A*02:01’, ‘HLA-A*68:02’, ‘HLA-A*26:01’, ‘HLA-A*32:01’]	FVYSFLMYL	0.09	ANTIGEN	Non-Toxin	Non-Allergen
[‘HLA-B*44:02’, ‘HLA-B*44:03’, ‘HLA-B*58:01’]	IDAMLNKRW	0.22	ANTIGEN	Non-Toxin	Non-Allergen
[‘HLA-B*08:01’]	KFDLQINSV	0.78	ANTIGEN	Non-Toxin	Non-Allergen
[‘HLA-B*57:01’, ‘HLA-B*58:01’]	LIDAMLNKRW	0.55	ANTIGEN	Non-Toxin	Non-Allergen
[‘HLA-A*02:01’, ‘HLA-A*02:03’, ‘HLA-A*32:01’, ‘HLA-A*02:06’]	LMYLLTPYL	0.27	ANTIGEN	Non-Toxin	Non-Allergen
[‘HLA-A*23:01’, ‘HLA-A*24:02’]	LMYLLTPYLL	0.25	ANTIGEN	Non-Toxin	Non-Allergen
[‘HLA-B*57:01’, ‘HLA-B*53:01’, ‘HLA-B*58:01’]	LTPYLLFRLW	0.19	ANTIGEN	Non-Toxin	Non-Allergen
[‘HLA-B*40:01’, ‘HLA-B*44:03’, ‘HLA-B*44:02’]	METELWPNL	0.07	ANTIGEN	Non-Toxin	Non-Allergen
[‘HLA-B*40:01’]	METELWPNLI	0.92	ANTIGEN	Non-Toxin	Non-Allergen
[‘HLA-A*23:01’, ‘HLA-A*24:02’, ‘HLA-A*32:01’, ‘HLA-B*08:01’]	MFMRFVYSF	0.04	ANTIGEN	Non-Toxin	Non-Allergen
[‘HLA-A*30:02’]	MRFVYSFLMY	0.91	ANTIGEN	Non-Toxin	Non-Allergen
[‘HLA-A*68:02’]	MTPTGSERV	0.23	ANTIGEN	Non-Toxin	Non-Allergen
[‘HLA-A*24:02’, ‘HLA-A*23:01’]	MYLLTPYLL	0.01	ANTIGEN	Non-Toxin	Non-Allergen
[‘HLA-A*23:01’, ‘HLA-A*24:02’]	MYLLTPYLLF	0.02	ANTIGEN	Non-Toxin	Non-Allergen
[‘HLA-A*68:01’, ‘HLA-A*33:01’, ‘HLA-A*31:01’]	NSVDRSQYR	0.06	ANTIGEN	Non-Toxin	Non-Allergen
[‘HLA-A*68:02’]	NTMVANASSV	0.43	ANTIGEN	Non-Toxin	Non-Allergen
[‘HLA-A*23:01’, ‘HLA-A*24:02’, ‘HLA-A*32:01’, ‘HLA-B*57:01’, ‘HLA-B*58:01’]	QYRELKSHW	0.15	ANTIGEN	Non-Toxin	Non-Allergen
[‘HLA-B*40:01’]	RESQNTMVA	0.47	ANTIGEN	Non-Toxin	Non-Allergen
[‘HLA-A*30:02’, ‘HLA-A*23:01’]	RFVYSFLMY	0.09	ANTIGEN	Non-Toxin	Non-Allergen
[‘HLA-A*32:01’]	RFVYSFLMYL	0.53	ANTIGEN	Non-Toxin	Non-Allergen
[‘HLA-A*02:06’]	SQNTMVANA	0.96	ANTIGEN	Non-Toxin	Non-Allergen
[‘HLA-B*15:01’, ‘HLA-A*30:02’]	SQYRELKSH	0.22	ANTIGEN	Non-Toxin	Non-Allergen
[‘HLA-A*32:01’, ‘HLA-B*57:01’, ‘HLA-B*58:01’, ‘HLA-B*44:03’, ‘HLA-B*44:02’, ‘HLA-A*23:01’, ‘HLA-A*24:02’, ‘HLA-B*15:01’]	SQYRELKSHW	0.27	ANTIGEN	Non-Toxin	Non-Allergen
[‘HLA-A*33:01’]	TGSERVKSR	0.53	ANTIGEN	Non-Toxin	Non-Allergen
[‘HLA-A*33:01’, ‘HLA-A*31:01’, ‘HLA-A*68:01’]	TMTPTGSER	0.34	ANTIGEN	Non-Toxin	Non-Allergen
[‘HLA-A*02:03’]	TMVANASSV	0.35	ANTIGEN	Non-Toxin	Non-Allergen
[‘HLA-B*53:01’, ‘HLA-B*51:01’, ‘HLA-B*58:01’, ‘HLA-B*57:01’, ‘HLA-B*35:01’]	TPYLLFRLW	0.03	ANTIGEN	Non-Toxin	Non-Allergen
[‘HLA-A*33:01’]	TPYLLFRLWR	0.75	ANTIGEN	Non-Toxin	Non-Allergen
[‘HLA-A*68:01’, ‘HLA-A*33:01’, ‘HLA-A*11:01’, ‘HLA-A*31:01’, ‘HLA-A*03:01’]	TTMTPTGSER	0.02	ANTIGEN	Non-Toxin	Non-Allergen
[‘HLA-A*02:01’, ‘HLA-A*02:03’, ‘HLA-A*02:06’]	VLEPIAMNV	0.07	ANTIGEN	Non-Toxin	Non-Allergen
[‘HLA-A*24:02’, ‘HLA-A*23:01’]	VYSFLMYLL	0.06	ANTIGEN	Non-Toxin	Non-Allergen
[‘HLA-B*15:01’, ‘HLA-A*02:06’, ‘HLA-B*40:01’]	YQDRESQNTM	0.08	ANTIGEN	Non-Toxin	Non-Allergen
**UM18 MHC** **1**					
**Allele**	**Peptide**	**Percentile Rank**	**Antigenicity**	**Toxicity**	**Allergenicity**
[‘HLA-B*51:01’]	DVMLVLLVI	0.44	ANTIGEN	Non-Toxin	Non-Allergen
[‘HLA-A*02:03’, ‘HLA-A*02:01’]	FMITAPMLT	0.78	ANTIGEN	Non-Toxin	Non-Allergen
[‘HLA-A*30:01’, ‘HLA-A*03:01’]	GQKLNVLVK	0.26	ANTIGEN	Non-Toxin	Non-Allergen
[‘HLA-A*23:01’, ‘HLA-A*24:02’]	IFMITAPML	0.25	ANTIGEN	Non-Toxin	Non-Allergen
[‘HLA-A*68:02’]	NVVPYIDVML	0.36	ANTIGEN	Non-Toxin	Non-Allergen
[‘HLA-A*23:01’, ‘HLA-A*24:02’]	PYIDVMLVL	0.05	ANTIGEN	Non-Toxin	Non-Allergen
[‘HLA-A*23:01’, ‘HLA-A*24:02’]	PYIDVMLVLL	0.15	ANTIGEN	Non-Toxin	Non-Allergen
[‘HLA-A*02:01’, ‘HLA-A*02:03’]	QLGSYFLNI	0.57	ANTIGEN	Non-Toxin	Non-Allergen
[‘HLA-B*51:01’, ‘HLA-B*53:01’]	VPYIDVMLV	0.01	ANTIGEN	Non-Toxin	Non-Allergen
[‘HLA-B*51:01’, ‘HLA-A*23:01’, ‘HLA-A*24:02’, ‘HLA-B*53:01’, ‘HLA-B*07:02’, ‘HLA-B*35:01’]	VPYIDVMLVL	0.19	ANTIGEN	Non-Toxin	Non-Allergen
[‘HLA-A*68:02’]	VVPYIDVML	0.85	ANTIGEN	Non-Toxin	Non-Allergen
[‘HLA-B*51:01’]	VVPYIDVMLV	0.43	ANTIGEN	Non-Toxin	Non-Allergen
[‘HLA-A*02:06’, ‘HLA-A*02:01’]	YIDVMLVLL	0.19	ANTIGEN	Non-Toxin	Non-Allergen

**Table 4:** List of IEDB-tool-generated peptides for MHC 2.

UMR5 MHC2							
Allele	Core Peptide	Peptide	Score	Rank	Antigenicity	Toxicity	Allergenicity
HLA-DRB1*15:01	IKLYQDRES	DAIIKLYQDRESQNT	0.9553	0.03	ANTIGEN	Non-Toxin	Non-Allergen
HLA-DRB4*01:01	LQINSVDRS	KFDLQINSVDRSQYR	0.8685	0.11	ANTIGEN	Non-Toxin	Non-Allergen
HLA-DRB3*02:02	IAMNVPVLS	LEPIAMNVPVLSGNQ	0.8766	0.11	ANTIGEN	Non-Toxin	Non-Allergen
HLA-DQA1*03:01/DQB1*03:02	IIAASTHDD	RPTIIAASTHDDEES	0.0446	0.12	ANTIGEN	Non-Toxin	Non-Allergen
HLA-DRB1*07:01	MTPTGSERV	VTTMTPTGSERVKSR	0.9229	0.12	ANTIGEN	Non-Toxin	Non-Allergen
HLA-DRB4*01:01	LQINSVDRS	MKFDLQINSVDRSQY	0.8112	0.14	ANTIGEN	Non-Toxin	Non-Allergen
HLA-DRB1*07:01	MTPTGSERV	LVTTMTPTGSERVKS	0.9045	0.15	ANTIGEN	Non-Toxin	Non-Allergen
HLA-DRB3*02:02	IAMNVPVLS	VLEPIAMNVPVLSGN	0.8152	0.16	ANTIGEN	Non-Toxin	Non-Allergen
HLA-DRB1*04:05	FDLQINSVD	NMKFDLQINSVDRSQ	0.9324	0.17	ANTIGEN	Non-Toxin	Non-Allergen
HLA-DRB3*02:02	IAMNVPVLS	EPIAMNVPVLSGNQV	0.7915	0.21	ANTIGEN	Non-Toxin	Non-Allergen
HLA-DRB1*04:05	FDLQINSVD	GNMKFDLQINSVDRS	0.9097	0.25	ANTIGEN	Non-Toxin	Non-Allergen
HLA-DRB1*07:01	MTPTGSERV	VLVTTMTPTGSERVK	0.8573	0.29	ANTIGEN	Non-Toxin	Non-Allergen
HLA-DQA1*01:02/DQB1*06:02	NASSVLESN	MVANASSVLESNKGS	0.7138	0.3	ANTIGEN	Non-Toxin	Non-Allergen
HLA-DRB1*09:01	YQLSVQAGF	QTVYQLSVQAGFNTG	0.7954	0.31	ANTIGEN	Non-Toxin	Non-Allergen
HLA-DQA1*04:01/DQB1*04:02	YRELKSHWG	DRSQYRELKSHWGED	0.3127	0.32	ANTIGEN	Non-Toxin	Non-Allergen
HLA-DRB1*15:01	IKLYQDRES	AIIKLYQDRESQNTM	0.8558	0.32	ANTIGEN	Non-Toxin	Non-Allergen
HLA-DRB4*01:01	LQINSVDRS	FDLQINSVDRSQYRE	0.6461	0.36	ANTIGEN	Non-Toxin	Non-Allergen
HLA-DQA1*03:01/DQB1*03:02	IIAASTHDD	PTIIAASTHDDEESQ	0.0299	0.47	ANTIGEN	Non-Toxin	Non-Allergen
HLA-DPA1*02:01/DPB1*05:01	RFQTVYQLS	HPERFQTVYQLSVQA	0.1584	0.5	ANTIGEN	Non-Toxin	Non-Allergen
HLA-DRB1*08:02	FLFKPVLNQ	KLKFLFKPVLNQFSG	0.785	0.51	ANTIGEN	Non-Toxin	Non-Allergen
HLA-DRB1*07:01	LVPVGGHNV	VGGSLVPVGGHNVLE	0.7903	0.52	ANTIGEN	Non-Toxin	Non-Allergen
HLA-DRB1*07:01	MTPTGSERV	TTMTPTGSERVKSRF	0.7883	0.53	ANTIGEN	Non-Toxin	Non-Allergen
HLA-DRB3*02:02	IAMNVPVLS	NVLEPIAMNVPVLSG	0.637	0.54	ANTIGEN	Non-Toxin	Non-Allergen
HLA-DPA1*02:01/DPB1*01:01	FQTVYQLSV	PERFQTVYQLSVQAG	0.2274	0.58	ANTIGEN	Non-Toxin	Non-Allergen
HLA-DRB1*04:05	FDLQINSVD	LGNMKFDLQINSVDR	0.8334	0.63	ANTIGEN	Non-Toxin	Non-Allergen
HLA-DRB3*02:02	LESNKGSVV	NASSVLESNKGSVVR	0.5862	0.66	ANTIGEN	Non-Toxin	Non-Allergen
HLA-DRB1*09:01	YQLSVQAGF	FQTVYQLSVQAGFNT	0.716	0.73	ANTIGEN	Non-Toxin	Non-Allergen
HLA-DRB1*08:02	FLFKPVLNQ	LKLKFLFKPVLNQFS	0.7158	0.84	ANTIGEN	Non-Toxin	Non-Allergen
HLA-DRB1*12:01	INSVDRSQY	DLQINSVDRSQYREL	0.6209	0.85	ANTIGEN	Non-Toxin	Non-Allergen
HLA-DRB3*02:02	MVANASSVL	ESQNTMVANASSVLE	0.4687	0.98	ANTIGEN	Non-Toxin	Non-Allergen
**UM1** **8**							
**Allele**	**Core peptide**	**Peptide**	**Score**	**Rank**	**Antigenicity**	**Toxicity**	**Allergenicity**
HLA-DRB1*11:01	LNVLVKGDQ	SGQKLNVLVKGDQGV	0.8626	0.6	ANTIGEN	Non-Toxin	Non-Allergen
HLA-DRB1*15:01	VVPYIDVML	SEINVVPYIDVMLVL	0.7499	0.7	ANTIGEN	Non-Toxin	Non-Allergen
HLA-DRB1*15:01	VVPYIDVML	ISEINVVPYIDVMLV	0.7017	0.93	ANTIGEN	Non-Toxin	Non-Allergen

## Data Availability

The original contributions presented in this study are included in the article/Supplementary Material. Further inquiries can be directed to the corresponding author.

## Supplementary Materials

The following supporting information can be downloaded at: https://www.mdpi.com/article/10.3390/ijms27187977/s1.
